# High Incidence of Esophageal Cancer in Women in Eritrea and Its Potential Link to Low Age at Menopause: Evidence from a 10-Year Retrospective Data Analysis

**DOI:** 10.1155/2024/5566016

**Published:** 2024-06-11

**Authors:** Samuel Tekle Mengistu, Yafet Kesete, Oliver Okoth Achila, Genet Tekeste Fikadu, Feven Abrhaley, Emnet Tekeste Fikadu, Salih Mohammed Said, Matiwos Araya Gheberehiwet, Mohammed Elfatih Hamida, Yosief Tewolde Ghidei

**Affiliations:** ^1^ Nakfa Hospital Ministry of Health Northern Red Sea branch, Nakfa, Eritrea; ^2^ Orotta College of Medicine and Health Sciences, Asmara, Eritrea; ^3^ Department of Pathology National Health Laboratory Ministry of Health, Asmara, Eritrea; ^4^ Barentu Military Hospital, Barentu, Eritrea; ^5^ Department of Microbiology National Health Laboratory Ministry of Health, Asmara, Eritrea; ^6^ Orotta College of Medicine and Health Sciences Unit of Microbiology, Asmara, Eritrea

## Abstract

**Background:**

Detecting a group of esophageal cancer (EC) cases in endemic regions is essential in identifying high-risk populations and executing appropriate interventions. The main aim of this study was to determine the epidemiology of EC in Eritrea.

**Methods:**

A retrospective (period: 2011 – 2021) study was carried out by abstracting data on EC patients from the logbook kept at the National Health Laboratory (ENHL). Information on socio-demographic, clinical history, and biopsy analysis findings was collected. For the statistical assessment of data, the End Results (SEER) Joinpoint Regression Program (V.4.5.0.1) was used to calculate crude incidence rate (CIR), age-adjusted incidence rate (ASR), and estimated annual percentage change (EAPC) by sex, age, and histotype.

**Results:**

A total of 189 patient's samples (134 (70.9%) females vs. 55 (29.1%) males, ratio 2.43 : 1) were evaluated. Of the 155 patients with EC, 44 (28.4%) and 111 (71.6%) were diagnosed with esophageal adenocarcinoma (EAC) and esophageal squamous cell carcinoma (ESCC), respectively (AC/ESCC ratio, 0.4). The median age (IQR) of patients with EC was 60 years (46.0 – 70 years) – (males 62 (IQR: 49.0 – 77 years) vs. females 60 (IQR: 46 -67 years), *p*-value =0.43. Within age bands, the F: M ratios in patients with ESCC were as follows: >20 -29 years =2: 1; 30-39 years =8 : 1; 40 – 49 years =10.5 : 1; 50-59 years =6.67 : 1; 60-69 years =3.25.1; 70-79 years =1.5 : 1 and>80 years =2 : 1. The all-age CIR and ASR for EC were 0.468 per 100 000 and 2.281 per 100 000 persons, respectively. Similarly, the all-age ASR for both males and females was 2.88 per 100 000 and 1.61 per 100 000. Over the study period, the EAPC for all cases was -3.0% (95% CI, −11.3 to 6.1, *p*-value =0.455).

**Conclusion:**

In large part, EC is a women's disease in Eritrea. The unusually high incidence of ESCC and the high female-to-male ratio point at sex-dependent exposures as a major driver of the EAC epidemic in the country. Therefore, research on the risk factors of EC in Eritrea is urgently needed.

## 1. Introduction

Esophageal cancer (EC) is a malignant disease that arises due to abnormal changes in the linings of the esophagus [1]. It is the 7^th^ most common and the 6^th^ leading cause of death among cancers worldwide [2]. Globally, incident cases, age-standardized incidence rates per 100 000 (ASR), estimated percentage in ASRs (EAPC) per 100 000, deaths, age-standardized mortality rates per 100 000 (ASMR), percentage in ASMR in 2019 for both sexes were 534 563 (95% uncertainty interval (UI): 466 513–595 343), 6.5 per 100 000 (95% UI: 5.7-7.3 per 100 000), -0.2% (95% UI: -0.3–0.0%), 498 067 (UI: 438 411-551 462), 6.1% (UI: 5.4-6.8), and -0.3 (-0.3-0.1) [2]. Further, this global burden of disease (GBD) report indicates that EC was responsible for 11.7 million (95% UI: 10.4-12.9 million) disability-adjusted life-years (DALY [2]. At the regional or national level, previous epidemiological studies suggest a highly heterogeneous picture of all these metrics. Importantly, experts agree that despite advances in diagnosis and treatment, the 5-year survival rate for EC is low (<20%), and the economic burden continues to increase [3]. The heavy toll is adequately captured in other epidemiological indexes such as years lived with disability (YLD) and years of life lost quality of life (YLL).

According to histotypes, dominant subtypes include esophageal squamous cell carcinoma (ESCC) (present in the upper two-thirds of the esophagus) and esophageal adenocarcinoma (EAC) (present in the lower third of the esophagus) [3, 4]. Rare ECs include neuroendocrine carcinoma, mesenchymal tumors, and lymphoma. Apart from disparate histological characteristics, ESCC and EAC differ in their clinical manifestation, pathogenesis, genetic risk profiles, and other risk factors [5]. Epidemiologically, ESCC is predominantly found in low-middle-income countries (LMICs) whereas EAC accounts for more than two-thirds of EC cases in high-income countries (HICs)—the United States (US), Australia, and Europe [6]. Globally, the CIRs of EAC continue to grow while ESCC has been declining [7, 8]. As a consequence of the dramatic rise in CIR of EAC in the last decade, it has been described as “an emerging disease” in HICs [7]. Socioeconomic index- (SDI-) backed geographical variance in the distribution of EC histotypes has been attributed to differential exposure to gender-dependent risk factors. For instance, the main risk factors for EAC in HICs are smoking, obesity (body mass index (BMI) ≥ 30 kg^2^), hypercholesterolemia, gastroesophageal reflux disease (GERD), especially erosive esophagitis, and its long-term *sequelae*, Barrett's esophagus [8–10]. In contrast, risk factors associated with ESCC include tobacco consumption (smoking and chewing tobacco), alcohol use, familial history of ESCC, unhealthy diet (drinking hot beverages, poor nutrition such as deficiency of vegetables and fruits, and presence of moldy/fermented foods or maize-based diet), indoor exposure biomass smoke, poor oral health (dental fluorosis), and medical condition treatments including achalasia, lye disease, Changes-associated megaesophagus, Plummer-Vinson syndrome, and repeated diagnostic radiation [5, 11, 12]. At present, the overriding presumption is that spatiotemporal disparities in the distribution of these risk factors can explain differences in EC (or ESCC) risk both within and between countries.

For reasons that are poorly understood, the ASIR of EC is disproportionately high in some parts of the world: the so-called “Asian esophageal cancer belt” (extends from Northern Iran, Central Asia, Mongolia, and North-Central China) and the “African esophageal cancer corridor” (extends from Ethiopia, Kenya to South Africa) [13]. In many ways, the true burden of EC, the African EC hotspot, is unknown. Previous reports have suggested that only 0.25% of sub-Saharan Africa (SSA) is covered by reliable death registries. High-quality data from population-based cancer registries (PBCRs) is also difficult to obtain. However, epidemiological studies along with recent reports from the WHO 2020 GLOBOCAN and IHME-GBD which rely heavily on covariate selection in models and regional patterns have provided some general information on the epidemiology. Regardless, experts agree that the high incidence of EC, particularly ESCC along the eastern corridor of Africa, is understudied [14, 15]. In the present study, we conducted an extensive epidemiological analysis of the burden of EC for both subtypes in Eritrea—a country in the “African esophageal cancer corridor.” The information generated can be used for health planning and allocations of resources.

### 1.1. Study Design and Setting

**Figure 1:**
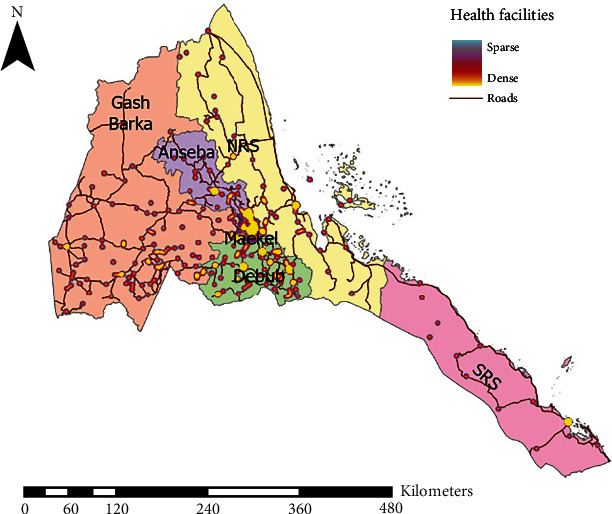
Geographical distribution of health services in Eritrea. A typical high concentration of health institutions in the central provinces of the country is shown.

This 10-year (January 2011–December 2021) institution-based retrospective study was undertaken by collecting laboratory data from the Eritrean National Health Laboratory (ENHL) (see Figure 1 for the distribution of health facilities in disparate administrative zones in the country). Besides having the only histopathological laboratory, ENHL has the only pathologists in the country. Therefore, all biopsy cases of esophageal tumors in the country requiring histopathological evaluation are processed at the facility. For the same reason, the laboratory register at the ENHL is the only repository where information on EC patients can be obtained. Data collected in the laboratory logbook include information on sociodemographic characteristics (age, sex, and address at time and date of specimen arrival), type and duration of clinical manifestations, primary site of malignancy (topography), physician impression, endoscopy outcomes, macroscopic and microscopic characteristics of specimen (histopathological morphology), degree of tumor differentiation, and final pathologist diagnosis. Note that registry data collection variables such as tumor length, depth of invasion, number of nodes involved, location of nodal disease, and presence of skip lesions (T (m)) among others, are not collected.

### 1.2. Tumor Classification and Case Definition

The International Classification of Diseases (ICD) definition of disparate histotypes of EC developed by the WHO was adopted in this study. The ICD-0 morphology codes for EAC were 8140–8573, and the related ICD-O codes for ESCC were 8050–8082 [16]. The laboratory employs the American Joint Committee on Cancer/Tumor-Node-Metastasis (AJCC/TNM) staging system [17], and diagnosis is per established protocol.

### 1.3. Data Collection/Retrieval

Patient information was collected from the ENHL logbook and digital database. To collect all the relevant information, a predesigned data collection form was used. Deidentification via the use of codes was used to maintain confidentiality. Males and females diagnosed with histopathologically confirmed EC in Eritrea were included in the study. For comprehensiveness and minimization of missing information and duplications, the data collected was cross-checked by 4 different enumerators.

### 1.4. Data Quality and Completeness

Data extracted from the laboratory records was subjected to consistency verification through analysis of age, sex, and histology. Incidence rate/year vs. age analysis was done to check data consistency and data stability. Moreover, data representativeness was evaluated using the residency of subjects. However, mortality-to-incidence ratio analysis was not undertaken due to a lack of data on mortality.

### 1.5. Data Analysis

Statistical data analysis was done using SPSS software version 26 (SPSS Inc., Chicago, USA), where appropriate, descriptive statistics, including frequencies/percentages, mean ± standard deviation (SD), and median + interquartile ranges, was employed. Chi-square (*χ*^2^) analysis was used to establish the degree of association between categorical variables. The information was presented using graphs and tables. Separately, we used the Joinpoint Regression Program (V.4.5.0.1) [18] to compute relevant epidemiological indexes (crude incidence rates (CIRs) (= new cases/year/100 000 population), age-standardized rates (ASR), estimated annual percentage change (EAPC), and associated error terms. In this analysis, a 10-year age band (<40, 40–49, 50–59, 60–69, and >70) was employed. For analysis of EAPC trend change points or Joinpoint segments, the program uses the following formula: exp (*β*) − 1] × 100 (where the regression coefficient (*β*) is an estimate of the linear regression between logarithmic-transformed age-standardized cancer rates per calendar year). Associated 95% confidence intervals (CIs) are typically generated from corresponding *t*-distribution function and regression analysis. All *p* values were considered to be of statistical significance when *p* < 0.05.

## 2. Results

### 2.1. Demographic Characteristics of Patients

From 2011 to 2021, a total of 189 samples (134 (70.9%) females vs. 55 (29.1%) males; ratio 2.43 : 1) were evaluated at the ENHL (Table 1). The all-sex median age (±IQR) was 60 (IQR: 46.50-70 years) (62 males (IQR: 49.0–77 years) vs. 60 females (IQR: 46-67 years)). Further, 23 (12.3%), 28 (15.0%), 32 (17.1%), 52 (27.8%), and 52 (27.8%) were in the <40, 40-49, 50-59, 60-69, and >70 years of age bands, respectively. Over the study period, 155 (82%) (42 (27.1%) males vs. 113 (72.9%) females) were histologically confirmed as EC. Although most patients were from the Central Region (76 (40.2%), histologically confirmed cases of EC were higher in regions outside the Central Region. In decreasing order, the most common symptoms are dysphagia (105 (54.4%)), weight loss (21 (10.9%)), vomiting/nausea (21 (10.9%)), epigastric pain (12 (6.3%)), and gastrointestinal bleeding (GI).

### 2.2. Esophageal Cancer Subtypes and Associated Factors

**Figure 2:**
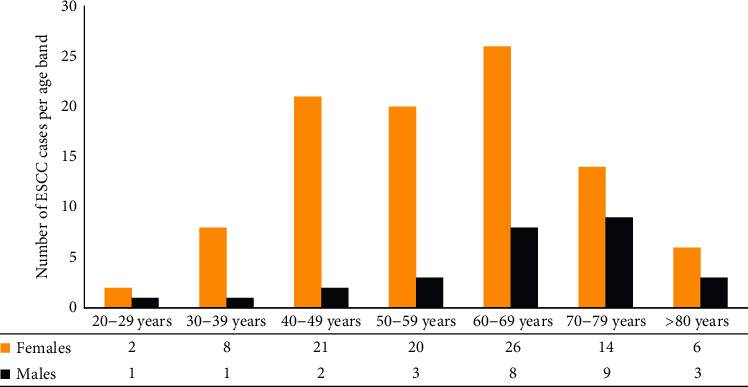
Sex-based distribution of ESCC cases per age band in EC patients in Eritrea, 2011-2022.

Of the 155 patients with EC, 44 (28.4%) and 111 (71.6%) were diagnosed with EAC and ESCC, respectively. The median age (IQR) of patients with EC was 60 years (46.0–70 years) (males: 5.0 (IQR: 51.25–77.75 vs. females: 60 years (IQR: 45–66 years), *p* value =0.43). The proportion of patients <45 years of age and >60 years of age was 25 (16.9%) and 83 (53.9%), respectively. Among patients with EC, 113 (72.9%) of the patients were females and 42 (27.1%) were males translating into a female-to-male count ratio of 2.69 : 1. Within age bands, the female-to-male count ratios in patients with ESCC were as follows: >20-29 years = 2 : 1; 30-39 years = 8 : 1; 40–49 years = 10.5 : 1; 50-59 years = 6.67 : 1; 60-69 years = 3.25.1; 70-79 years = 1.5 : 1; and >80 years = 2 : 1 (see Figure 2). Importantly, the female-to-male count ratio in EAC and ESCC was 1.06 : 1 and 3.48 : 1, respectively. In other words, ESCC in females was significantly higher—97 (85.8%) vs. 27 (64.3%) in males (*p* value =0.005) (see Table 2 for additional details).

Separately, we observed that the median (IQR) of symptoms was higher in patients with EAC, 4 months (3–4 months) vs. 3 months (2-5 months) in patients diagnosed with ESCC, *p*-value =0.006. Regarding the histopathological subtype differentiation, 124 (80.0%; 95 CI: 72.9-86.5) cases were diagnosed with ESCC and 31 (20.0%; 95 CI: 13.5-27.1) were diagnosed with EAC. Furthermore, 32 (20.6%) of the patients had well-differentiated tumors, 34 (21.9%) had moderately differentiated (grade 2 malignancy) tumors, and 43 (27.7%) patients had poorly differentiated tumors. However, 46 (29.6%) of the cases had no conclusive reports on their tumor differentiation grade. Lastly, a significant proportion of patients with EAC was from the Central Region (20 (66.7%)). The dominant subtype in the other regions was ESCC.

### 2.3. Crude Incidence Rate (CIR), Age-Standardized Incidence Rates (ASR), and Estimated Annual Percentage Changes (EAPC) of Esophageal Cancer (EC) in Males and Females in Eritrea, 2011-2021

The ASR, CIR, and EAPC for all EC cases are demonstrated in Table 3. The all-age CIR and ASR for EC were 0.468 per 100 000 and 2.281 per 100 000 persons, respectively. Similarly, the all-age ASR for both males and females was 2.88 per 100 000 and 1.61 per 100 000. Over the study period, the EAPC for all cases was -3.0% (95% CI, −11.3 to 6.1, *p* value =0.455). The cumulative CIR (ASR) males vs. females were 0.26 per 100 000 (1.61 per 100 000) vs. 0.67 per 100 000 (2.88 per 100 000), respectively. Associated EAPC in males and females were -11.4 (95% CI: -21.5 to 0.1), *p* value 0.051, and 1.3 (95% CI: -7.1 to 10.4), *p* value =0.742.

### 2.4. Crude Incidence Rate (CIR), Age-Standardized Incidence Rates (ASR), and Estimated Annual Percentage Changes (EAPC) Associated with Different Histotypes of Esophageal Cancer (EC)

The cumulative CIR (ASR) for ESCC and EAC was 0.358 per 100 000 (0.999 per 100 000) and 0.084 per 100 000 (0.343 per 100 000), respectively (Table 4). During the study period, the EAPC for ESCC and EAC was -11.2% (95% CI, -23.2 to 2.6, *p* = 0.09) and 6.6% (95% CI, -13.8 to 32, *p* value =0.507). In this period, the overall ratio of ESCC: EAC was 4 : 21 (see Table 4 for additional information).

## 3. Discussion

In the context of a rapid rise in CIR of cancers in older adults from SSA, our study describes the CIR, ASR, and EAPC of EC in Eritrea. Few epidemiological studies of EC, including assessment of the predominant histotypes, have been conducted in the country. As a consequence, the epidemiology of the disease is poorly understood. The principal findings were (i) a high CIR and ASR of EC relative to that reported in other countries in the region, (ii) a stable all-sex EAPC, (iii) a highly unusual high female-to-male ratio through the study period, (iv) a high frequency of ESCC, and (iv) a predominance of cases (particularly EAC) from the Central Region. Other notable results include the high number of patients <40 years, and a high number of late presentations (unresectable or metastatic disease).

From among 204 countries and territories, a recent GDB report on the burden of EC noted that some of the highest ASR in SSA were recorded in Malawi (24.5 per 100 000 (95% UI: 18.7-32.5 per 100 000)) and Uganda (15.6 per 100 000 (95% UI: 12.1–19.5 per 100 000)) people [19], and Zimbabwe (15.4 per 100 0000). Although, the current 10-year ASR estimate of 2.54 per 100 000 people reported in this study is low; it is higher than comparable estimates for Nigeria (1.0 per 100 000 (95% CI: 0.7–1.8 per 100 000)), Tunisia (0.96 per 100 000), and Algeria (1.08 per 100 000) [2]. Importantly, the estimates are relatively similar to those reported for Ethiopia, 2.7 per 100 000 (95% CI: 2.1-3.7 per 100 000) persons [20].

As reported in previous studies of cancers in Eritrea, we believe that the ASR reported in this study is likely to be an underestimation. Underdiagnosis and underdocumentation/underreporting (deficit between actual and reported incidences) are potential sources of underestimation in retrospective observational studies. Underdiagnosis is mostly a by-product of the well-documented deficiencies in laboratory systems and health delivery components such as management of health information [15]. As previously stated, Eritrea has a single laboratory where all EC biopsy specimens are processed, and most imaging centers are located in Asmara—note that computer tomography (CT), positron emission PET/CT, and endoscopic ultrasound- (EUS-) guided fine needle biopsy are not available. Due to the lack of a formal infrastructure for the transport of biopsy specimens from other hospitals in the country, patients are referred in person to referral facilities in Asmara. Predictably, only a fraction of patients will travel to Asmara due to the long distances and associated out-of-pocket costs. This problem is further compounded by poor referral practices. In our analysis, these assumptions were evidenced by the disproportionate number of EC patients (40.2%) from the Central Region. In contrast, 4 (2.1%) patients were from the Southern Red Sea, a subzone with some of the remotest regions in Eritrea. This notwithstanding, substantial within-country variation in ASR for EC or within subgroups is a well-documented phenomenon [21]. In this context, what may be interpreted as underdiagnosis and underreporting from other subzones may be a true reflection of geographical differences in incidence. While plausible, we believe that this possibility is highly unlikely.

Importantly, we noted that the EAPCs remained stable (-3.0% (CI: -11.3 to 6.1), *p* value =0.455) over the study period. Disaggregation of EAPC data between the sexes demonstrated a more subtle result with a declining trend in males that tended towards significance (-11.4% (95% CI: -21.5 to 0.1), *p*-value 0.051 in males). Temporal trends in ASR of EC tend to vary considerably. From 1990 to 2019, for instance, 119 countries experienced a significant decrease in ASR [2]. In men, drastic decreases in EAPC were reported in Brazil (-5.94 (95% CI: -10.44 to -1.21)) and India (-5.00 (95% CI: -8.18 to -1.70)) [22]. In contrast, a small number of countries (58) reported increasing trends, while others reported relatively stable epidemics. The reason behind the decrease in EAC in some populations has been attributed to changing lifestyles, particularly the reduction in the consumption of tobacco by men [13].

As previously reported by multiple investigators in the region, ESCC was the predominant histotype [22, 23]. Our analysis also demonstrated that the 10-year ASR of EAC was relatively low, at 3.43 per 100 000. According to studies, low SDI is associated with a high incidence of ESCC, and high SDI is associated with a high incidence of EAC [2]. This pattern has been linked to exposure heterogeneity particularly the spatiotemporal distribution of the main risk factors of EAC or ESCC. In our data, the incidence of EAC was disproportionately high in the Central Region, where Asmara, the capital city of Eritrea, is located. Interestingly, past reports have suggested that Asmara has a high incidence of obesity (high BMI and waist circumference) and metabolic syndrome [24]—the main risk factors for EAC [9, 10]. By itself, this outcome highlights the possibility that EAC may become an increasingly important contributor to the future burden of EC in the country. To address this concern, it is critical to monitor and potentially slow down the rising or high incidence of obesity and metabolic syndrome in the country.

Regarding the excess ESCC burden observed in the female population, we have to emphasize that this outcome is highly unusual. In most part, multiple studies of EC epidemiology indicate a predominance in men—388, 827 (95 CI: 335 510–444 000) in men vs. 145 736 (95% CI: 119 952–165 068) in females, ratio 2.66 : 1 [2]. A wide between-country variation in male-to-female ASR ratios has also been reported for EC: ratios > 10 : 1 in several Baltic and European countries (Belarus, Slovenia, Lithuania, and Ukraine), 4.79 (95% CI: 4.71-4.87) in the USA, 1.58 (95% CI: 1.50-1.66) in India [25], 1.4 in South Africa [26], and 1.4 to 2 across Africa [21]. In this study, the male-to-female count ratio was inverted, 1 : 2.69. The results are consistent with ASR ratios for ESCC (3.8 per 100 000/4.5 per 100 000) reported in a recent study [25]. Another equally remarkable observation was that the inverted male-to-female ratio was consistent across time periods and that this difference was mostly observed for ESCC (1: 1.06 for EAC vs. 1: 3.48 for ESCC). A similar inversion (excess ESCC in females) has been reported in Djibouti [27], Sudan [28], and Ethiopia [25, 29]. Highlighting the global variation in male-to-female ratios in ESCC, some researchers have argued that the ratio tends to be lower in LMIC, approaching or even exceeding 1 : 1 [13, 21].

In contrast to EAC, previous studies from China, the US, Europe, and some parts of Africa demonstrate considerable etiologic heterogeneity for ESCC [14, 21]. Predictably, male dominance has been attributed, in large part, to gender-patterned exposures including differential disparities in sex steroid hormone activity, differences in health-seeking behavior or gender referral biases, and differences in time- or trend-dependent environmental exposures (alcohol and tobacco), among others [21, 30]. In Eritrea, the potential contribution of some of these exposures to the high burden of EC or sex differences in ESCC is difficult to discern due to a lack of data. However, the contribution of some known risk factors to the observed outcome is easy to dismiss. For instance, we believe that tobacco consumption, oral hygiene, and population sex imbalances may contribute little (or not at all) to the high incidence of ESCC among women in the country. This is because tobacco consumption among women is minimal. Although data on secondary exposure of women is usually not provided in most studies, there is a consensus that tobacco consumption is higher in males [21].

On the contrary, there is some indication that environmental chemical exposures (biomass smoke, nitrosamines, and mycotoxins) or in-house pollution can be higher for women in Eritrea. This position is supported by prior studies that reported that exposure to biomass smoke can be higher for females due to time spent cooking indoors in kitchens with poor ventilation [30]. In one such study, Mwachiro et al. demonstrated that urinary concentrations of polycyclic aromatic hydrocarbons (PAHs) were strongly associated with indoor cooking with wood [31]. They also demonstrated a significant association between 2-hydroxy naphthalene and histologic esophageal squamous dysplasia (ESD)—a premalignant lesion for ESCC.

Although biomass smoke is emerging as an important contributor to ESCC risk in the “African esophageal cancer corridor,” much remains to be studied. For example, the possibility that trees from disparate geographical regions may have different volatile compounds when combusted and that the risk profiles of these compounds can also be different is unexplored. Further, consumption of traditional beer (*Siwa*—the brew is typically mixed with extracts from *Rhamnus prinoides*) is relatively widespread among women in some parts of Eritrea. In a large-multicentre study, some researchers recently stated that alcohol consumption has one of the largest population-attributable fractions (PAF) for ESCC in Kenya, Tanzania, and Malawi [6]. However, in this setting, we do not believe that sex differences in alcohol consumption can explain the observed female excess. A similar position can be taken with regard to socioeconomic status, consumption of hot beverages (tea and coffee > 65° Celsius), population sex ratios, referral biases, and other understudied exposures such as deficiencies in iron (Fe), zinc (Zn), magnesium (Mg), and selenium (Se) [13, 32, 33]. Regardless, future investigations into these associations are needed.

Regarding hormonal differences, we will argue that if the typical male dominance in ESCC prevalence before the 60–70-year age band is significantly influenced by estradiol levels as some suggest, then our results (or results from northeastern parts of SSA) countermand this suggestion. As previously described, the incidence of ESCC was higher in the age range (40–59 years). In the >60-year age band (e.g., postmenopausal period), female-to-male ratios were largely comparable. To explain these findings, we need to deal with several explanations. First, we can argue, with some merit, that the highlighted ratios are count ratios which may be subject to heavy fluctuations given the limited number of cases in some age bands. Secondly, one can also assert that the role of steroid sex hormones in predisposition to higher ESCC risk may be marginal and, as previously noted, that geographically restricted, sex-dependent environmental exposures play a dominant role in most settings. Abnet et al. justified this position by noting that the association between estrogen levels and ESCC may be due to nonhormonal factors or confounding by socioeconomic status [13]. This argument is generally countermanded by a relatively common observation that dominant risk factors such as alcohol and tobacco cannot fully explain male predominance in EC (EAC or ESCC) [21].

**Figure 3:**
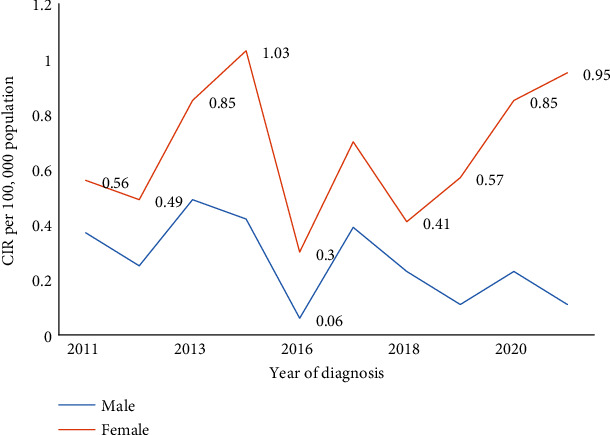
Crude incidence rate of esophageal cancer among sexes, 2011–2021, Eritrea. Data from 2015 was not included in the table because no samples were processed in the laboratory that year.

An equally plausible explanation for the results observed in this setting connects the hypothesis regarding the protective role of estrogen and population level prevalence of early menopause (menopause < 45 years of age). Here, the underlying argument is that if a link between low levels of estrogen and augmented ESCC risk exists, then there should be a link between early menopause (menopause is associated with serum oestradiol levels) and increased risk of ESCC in some populations. It also follows that the low mean or median age at menopause coupled with the preponderance of some differentiating risk factors in females (known or unknown) should lead, either to a smaller difference in a male-to-female ratio (a phenomenon observed in a large number of countries in Eastern and southern parts of Africa) or to a female gender excess in some populations. In other words, PAF of ESCC due to early menopause may be large in some populations and this may have a significant influence on sex ratios. Partly based on this logic, we believe that the results displayed in Figure 3 (or the inverted sex ratio) can be explained by the invocation of early menopause. Supporting this position is the observation that a large number of countries with female gender bias in ESCC ASR have a relatively low mean or median age at menopause: 45.23 years (or lower) in Ethiopia [34], 45 (±3.4) years in Eritrea [35], and 43 (±3) in Sudan [36]—note that the data referenced here is mostly regional.

Although the link between low levels of estrogen and EC or ESCC has been explored by a relatively small number of inconclusive studies—none of which is from SSA—direct and indirect evidence of a connection exists. For instance, The Million Women Study (MWS) cohort study demonstrated a strong and consistent association between elevated risk of ESCC and age at menopause (relative risk (RR), 95% CI per 5 years younger at menopause, 1.32, 1.11–1.56) [37]. McCarthy et al. noted a two-fold risk elevation of ESCC risk in women entering menopause at age < 45 years (vs. those entering menopause at age > 50 years) [38]. Further supporting a role from estrogen in ESCC etiology are findings showing a decreased esophageal ESCC risk (hazard ratio (HR) = 0.25, 95% CI: 0.28-3.26) in women on estrogen + progestin (*E* + *P*) therapy [39]. Utsimu et al. reported on the in vitro inhibition of estrogen receptor (ER) positive (+) ESCC cell lines (ES-25C) with 17*β*-estradiol and an inverse relationship between plasma level of estrogen and EAC [40]. In addition, Wang et al. noted that hormone replacement therapy (HRT) was negatively associated with ESCC risk (pooled RR = 0.68, 95% CI, 0.48-0.97, *p* = 0.031) [41] whereas recent work by Xie et al. corroborated these findings by highlighting a connection between HRT (estrogen only or estrogen + progestogen) and reduced risk of EC [42]. With varying consistency, others have found evidence of a link between ESCC and HRT [43, 44]. The highlighted connection between estrogen deficiency (e.g., in early menopause) and high ESCC risk should be noted and is not at variance with the possibility of a direct causal connection between dominant exposures such as tobacco or alcohol–related carcinogens and ESCC in women. It merely offers an alternative causal pathway for multiple agents.

Etiological clues on the contribution of low age at menopause on ESCC epidemiology can further be gained from the fact that the two disorders share a large number of risk factors: tobacco consumption, alcohol use, poor nutrition, breastfeeding, BMI, family history, and socioeconomic status (SES) [12, 26, 45], among others. Respecting the temporal sequence of exposures in both early age at menopause and ESCC, data shows that they operate in similar timeframes in the life course. For example, Luebeck et al. noted that early childhood exposures such as pediatric malnutrition, vitamin deficiencies (thiamin (vitamin B_1_) and riboflavin (vitamin B_2_)), and exposure to specific pollutants, among others, may produce premalignant field defects associated with ESCC [46]. These young-age ESCC-predisposing signatures, later argued, can be aggravated by differentiating risk factors later in life. Recently, analysis of data from a long-running population-based prospective cohort study in Britain demonstrated that similar time- or trend-dependent environmental exposures are associated with early menopause [44]. Similarly, McCormack et al. argued that the low mean age of EC patients in parts of SSA may be driven by environmental agents acting from early in the life course, in addition to inherited susceptibility [30]. In the end, we postulate an alternative causal pathway where the shared risk factors act through early menopause to augment ESCC risk in women.

From the foregoing discussion, it is clear that the concept of estrogen deficiency (in early menopause) as a risk for ESCC or its impact on EC epidemiology in the overall population deserves further exploration. Further, it is evident that research on context-specific factors associated with ESCC in females in countries like Eritrea (or other countries in the “African esophageal cancer corridor”) should ideally take a life course approach that focuses on exposures at birth/early life, childhood, early adulthood, and late adulthood. Researching the temporal ordering of potential risk *via* multisite large-scale population-based prospective cohort studies that incorporate a life course approach can help identify differences in exposure patterns by age, latency of effects, or other complex interactions [47]. Apart from expanding the range of variables (e.g., *intrauterine* exposures and reproductive hormones, among others) in ESCC risk research, a life course approach can also help identify critical periods where susceptibility to etiological agents is most harmful [45]. In this regard, it can provide information on timely interventions and clarify many of the contradictions found in EC risk factor literature.

And lastly, evidence of an inherited component to the ESCC and EAC etiologies exists. In a study, utilizing SNP-, gene-, and pathway-based associations, Hyland et al. uncovered a connection between ESCC risk and genetic variation in sex hormone metabolic pathways [48]. Among the myriad candidate lesions, mutations associated with functional variants in alcohol-metabolizing genes, ALDH2 and ADH1B [49]; phospholipase c epsilon 1 gene (PLCE1); p53 [50] and p16/CDKN2 genes; and RUNX1 rs2014300 [12] and VSIG10L [51, 52], among others, have been implicated. The well-documented association between EC and RHBDF2 gene at 17q25 in tylosis (a rare genetic disease characterized by focal nonepidermolytic palmoplantar keratoderma) adds to the evidence [13]. In Malawi, whole-exome sequencing of 59 ESCC tumors uncovered tumor mutation signatures that were described as possibly consistent with an unknown carcinogenic exposure [53]. Further, family history, a marker of genetic susceptibility or common environmental exposures, has been linked to EC in the past [54]. Apart from shared cultural practices, some ethnic groups in Eritrea, Djibouti, Ethiopia, and parts of Sudan share a common ancestry. As noted previously, all these countries have an inverted male-to-female ESCC ratio, a relatively low mean age at menopause, and an unusually high proportion of young patients (<45 years) with ESCC [55]. These commonalities strongly suggest that shared genes may underpin the development of ESCC in these populations. Unfortunately, our understanding of the etiology and molecular mechanisms involved in EC in Eritrea is limited. Whatever the root causes of the high ASR or the female gender excess in Eritrea, much work is needed to understand the contribution of genetic and nongenetic factors in ESCC risk.

### 3.1. Limitation

Some limitations in this study must be mentioned. First, retrospective studies are undermined by multiple problems associated with data quality—item missingness, questionable robustness of data collection approaches, and data quality evaluation, among others. Secondly, we believe that the problem of underdiagnosis and underreporting was extremely severe in some subzones. Because we did not undertake any adjustment to mitigate this concern; our estimates are highly conservative. Thirdly, we will concede that even though ASR is a useful metric in epidemiological studies involving cancers, its reliability may be compromised in countries with relatively small populations like Eritrea due to small sample sizes and the potential for random fluctuations. This can result in large 95% CIs and type II error rates. In our study, this drawback was underscored by the small number of EAC cases. Lastly, misclassification of tumors is a possibility in this setting. On this issue, investigators have pointed at the challenge involved in differentiating between ESCC and gastric cardia adenocarcinoma in settings with a high burden of EC given the absence of a definitive criterion for identifying either lesion in adenocarcinomas than span the esophagogastric junction. These notwithstanding, we believe that the approach used in this study is superior to alternatives which rely exclusively on mathematical modelling or extrapolations from neighboring countries.

## 4. Conclusion

Elucidating the gender differences between specific risk factors is a key component of ongoing research on ESCC etiology in SSA. While male excess in ESCC incidence is well evidenced, research on abnormally high ASR in males and/or female excess has been reported in some countries. In Eritrea, for example, the ASR of EC is slightly high with a lop-sided burden in women. The high female-to-male ratio points at the potential existence of geographically distinct, sex-dependent exposures that appear to act early in the life course. To explain our finding, we posited that cohort and periodic effects, acting through early menopause, may partly account for the observed outcome. Overall, etiologic research priorities (including by the African Esophageal Cancer Consortium (AfrECC)) for ESCC in countries with females excess or low male-to-female ESCC ASR ratios need to take this observation seriously. Separately, our result points at the need for increased cancer prevention and control effort. In this regard, prioritizing EC in national cancer control plans is clearly important. Additional strategies include the establishment of PBCRs and a robust cancer surveillance system to collect, collate, and investigate cancer cases in the country. On the other hand, access to cancer diagnostics can be improved by a three-pronged approach: an efficient and coordinated biopsy or patient referral system at all levels of care, improvements in diagnostic capacity of referral facilities, and establishment of pathology laboratories (for processing of biopsy specimen for subsequent evaluation by the pathologist at the ENHL) at regional referral hospitals in the country.

## Figures and Tables

**Table 1:** Characteristics of esophageal malignancies across risk factors, Eritrea, 2011-2021.

Characteristics	Nonmalignant disease *N* (%)	Malignant disease *N* (%)	*p* value (*χ*^2^)	Total *N* (%)
Age in years	57 (47.25-70)	60 (45.75-70)	0.89^a^	60 (46.75-70)
<40	7 (21.21)	16 (10.4)	0.15 (6.75)	23 (12.3)
40-49	2 (6.1)	26 (16.9)		28 (15.0)
50-59	3 (9.1)	29 (18.8)		32 (17.1)
60-69	11 (33.3)	41 (26.6)		52 (27.8)
70+	10 (30.3)	42 (27.3)		52 (27.8)
Gender				
Male	13 (38.3)	42 (27.1)	0.19 (1.67)	55 (29.1)
Female	21 (61.8)	113 (72.9)		134 (70.9)
Address				
Gash Barka	0 (0)	22 (14.3)	0.01 (14.8)	22 (11.6)
Southern	7 (20.0)	34 (22.1)		41 (21.7)
Central	23 (65.7)	53 (34.4)		76 (40.2)
Anseba	1 (2.9)	12 (7.8)		13 (6.8)
Southern Red Sea	0 (0)	4 (2.6)		4 (2.1)
Northern Red Sea	4 (11.4)	29 (18.8)		33 (17.4)
Symptom duration in months (median ± IQR)	4 (3-9)	3 (2.6-5)	0.782 (0.07)	3 (3-5)
Symptoms/indications for EGD				
Dysphagia	11 (68.8)	94 (89.53)	0.094 (2.81)	105 (54.4)
Vomiting/nausea	5 (26.3)	16 (11.1)	0.62 (0.25)	21 (10.9)
Epigastric pain	1 (5.3)	11 (7.6)	0.31 (1.04)	12 (6.2)
Weight loss	1 (5.3)	20 (13.9)	0.06 (3.32)	21 (10.9)
GI bleeding	1 (5.3)	3 (2.1)	0.78 (0.07)	4 (2.1)

Abbreviations: EGD: esophagogastroduodenoscopy. ^a^Independent sample Mann–Whitney *U*-test.

**Table 2:** Characteristics of types of esophageal cancer in comparison to risk factors, Eritrea, 2011-2021.

Characteristics	EAC *N* (%)	ESCC *N* (%)	*p* value (*χ*^2^)	Total *N* (%)
Age (mean ± SD)	62 (50-70)	60 (45-70)	0.43^a^	60 (46.75-70)
<40	5 (16.7)	12 (9.7)	0.58 (2.81)	17 (11.0)
40-49	3 (10)	23 (18.5)		26 (16.9)
50-59	5 (16.7)	23 (18.5)		28 (18.2)
60-69	7 (23.3)	34 (27.4)		41 (26.6)
70+	10 (33.3)	32 (25.8)		42 (27.3)
Gender				
Male	14 (46.7)	27 (21.6)	0.005 (7.81)	41 (26.5)
Female	16 (53.3)	98 (78.4)		114 (73.5)
Symptom duration in months (median ± IQR)	4 (3-4)	3 (2-5)	0.006 (8.22)	3 (3-5)
Address				
Central	20 (66.7)	33 (26.6)	0.0005 (21.7)	53 (34.4)
Southern	5 (16.7)	29 (23.4)		34 (22.1)
Gash Barka	0 (0)	21 (16.9)		21 (13.6)
Anseba	3 (10)	10 (8.1)		13 (8.4)
Northern Red Sea	1 (3.3)	28 (22.6)		29 (18.8)
Southern Red Sea	1 (3.3)	3 (2.4)		4 (2.6)
Grade				
Well differentiated	0 (0)	32 (33.7)	0.016 (8.23)	32 (29.4)
Moderately differentiated	8 (57.1)	26 (27.4)		34 (31.2)
Poorly differentiated	6 (42.9)	37 (38.9)		43 (39.4)

Abbreviation: EAC: esophageal adenocarcinoma; ESCC: esophageal squamous cell carcinoma. ^a^Independent Mann–Whitney *U*-test.

**Table 3:** Crude and age-adjusted incidence rates of esophageal cancer, Eritrea, 2011-2021.

Year	Count	Total	Count	Male	Count	Female	M : F ratio
		CIR (ASR)		CIR (ASR)		CIR (ASR)	
2011	15	0.46 (2.82)	6	0.37 (2.62)	9	0.56 (3.02)	0.67
2012	12	0.37 (1.91)	4	0.25 (1.46)	8	0.49 (2.34)	0.5
2013	22	0.67 (3.75)	8	0.49 (3.17)	14	0.85 (4.30)	0.57
2014	24	0.73 (2.91)	7	0.42 (2.31)	17	1.03 (3.45)	0.41
2016	6	0.18 (0.62)	1	0.06 (0.38)	5	0.30 (0.89)	0.2
2017	19	0.54 (2.34)	7	0.39 (2.12)	12	0.70 (2.63)	0.58
2018	11	0.32 (1.83)	4	0.23 (1.61)	7	0.41 (2.01)	0.57
2019	12	0.34 (1.62)	2	0.11 (0.43)	10	0.57 (2.46)	0.2
2020	19	0.54 (2.44)	4	0.23 (1.21)	15	0.85 (3.43)	0.27
2021	19	0.53 (2.60)	2	0.11 (0.75)	17	0.95 (4.24)	0.12
Mean	15.9	0.47 (2.28)	4.5	0.26 (1.61)	11.4	0.67 (2.88)	0.39
EAPC		-3.0		-11.4		1.3	
95% CI		-11.3 to 6.1		-21.5 to 0.1		-7.1 to 10.4	
*p* value		0.455		0.051		0.742	

Abbreviations: EAPC: estimated annual percent change; M : F ratio: male-to-female ratio. Data from 2015 was not included in the table because no samples were processed in the laboratory that year.

**Table 4:** Trend of crude and age-adjusted rate per 100 000 by year for each histotype of esophageal cancer, Eritrea, 2011-2021.

Year of diagnosis	Count	EAC		Count	ESCC		ESCC : EAC ratio
		CIR	ASR		CIR	ASR	
2011	1	0.03	0.18	11	0.34	1.54	11.00
2012	3	0.09	0.45	9	0.28	0.76	3.00
2013	3	0.09	0.59	18	0.55	2.66	6.00
2014	6	0.18	0.21	17	0.51	1.14	2.83
2016	1	0.03	0.02	5	0.15	0.38	5.00
2017	3	0.08	0.36	16	0.45	1.14	5.33
2018	2	0.06	0.35	9	0.26	1.14	4.50
2019	1	0.03	0.18	10	0.29	0.09	10.0
2020	4	0.11	0.37	15	0.42	0.75	3.75
2021	5	0.14	0.72	12	0.33	0.39	2.40
Mean	2.9	0.084	0.343	12.2	0.358	0.999	4.21
EAPC		6.6			-11.2		
95 (CI)		-13.8 to 32			-23.2 to 2.6		
*p* value		0.507			0.095		

Abbreviation: EAC: esophageal adenocarcinoma; ESCC; esophageal squamous cell carcinoma; EAPC: estimated annual percent change. Data from 2015 was not included in the table because no samples were processed in the laboratory that year.

## Data Availability

The datasets supporting the conclusions of this article are available as part of the manuscript.
